# Phylogenetic diversity meets conservation policy: small areas are key to preserving eucalypt lineages

**DOI:** 10.1098/rstb.2014.0007

**Published:** 2015-02-19

**Authors:** Laura J. Pollock, Dan F. Rosauer, Andrew H. Thornhill, Heini Kujala, Michael D. Crisp, Joseph T. Miller, Michael A. McCarthy

**Affiliations:** 1 School of Botany, The University of Melbourne, Parkville, Victoria, Australia; 2 Research School of Biology, Australian National University, Canberra, Australian Capital Territory, Australia; 3 Australian Tropical Herbarium, James Cook University, Cairns, Queensland, Australia; 4 Australian National Herbarium, CSIRO, Plant Industry, Canberra, Australian Capital Territory, Australia

**Keywords:** Australia, *Eucalyptus*, phylogenetic diversity, reserve selection, species distribution modelling, spatial prioritization

## Abstract

Evolutionary and genetic knowledge is increasingly being valued in conservation theory, but is rarely considered in conservation planning and policy. Here, we integrate phylogenetic diversity (PD) with spatial reserve prioritization to evaluate how well the existing reserve system in Victoria, Australia captures the evolutionary lineages of eucalypts, which dominate forest canopies across the state. Forty-three per cent of remaining native woody vegetation in Victoria is located in protected areas (mostly national parks) representing 48% of the extant PD found in the state. A modest expansion in protected areas of 5% (less than 1% of the state area) would increase protected PD by 33% over current levels. In a recent policy change, portions of the national parks were opened for development. These tourism development zones hold over half the PD found in national parks with some species and clades falling entirely outside of protected zones within the national parks. This approach of using PD in spatial prioritization could be extended to any clade or area that has spatial and phylogenetic data. Our results demonstrate the relevance of PD to regional conservation policy by highlighting that small but strategically located areas disproportionally impact the preservation of evolutionary lineages.

## 1. Introduction

The value of including evolutionary information in conservation has been well established, but evolutionary diversity is rarely considered in policy and management [1,2]. Using ancestral relationships when selecting species for conservation was suggested more than 20 years ago [3–5]. The essence of the argument is that species should be valued based on their contribution to the tree of life. The evolutionary contribution of taxa is most commonly measured by phylogenetic diversity (PD) or the length of the shared pathway on a phylogeny represented by a set of taxa [5]. A large body of literature has since developed around several PD related subtopics, and the use of PD has reached fields as diverse as community ecology [6] and bioprospecting [7]. The uptake of PD into applied conservation has lagged behind the literature, but PD-type metrics are now being used to rank global species with the evolutionarily distinct globally endangered (EDGE) list [8] and assigning regional conservation priorities for species [9] and areas [10].

One of the arguments for why PD is not more fully integrated in conservation is that PD is not always a surrogate for other conservation values [1], but conserving PD is a goal in itself if we value biodiversity in conservation [11]. There are many additional benefits of retaining the widest possible portion of the tree of life. Conservation scenarios with PD effectively select medically and economically important plants in the Cape of South Africa [12]. The bioactive compounds in current use are so diverse that it would be difficult to pinpoint which types will be important in the future [13]. For example, in eucalypts, a diversity of potentially useful chemistry exists beyond the small subset of species and compounds currently used in products ranging from cough suppressants to insecticides [14]. Even in this relatively well-studied and commercially important plant group, new classes of chemicals with potential for therapeutics, including cancer treatment, are actively being discovered [15]. Given less than 15% of plant species have been screened for bioactivity [16], many useful but unknown compounds probably exist. Preserving PD increases our ‘option values’ [17]—the likelihood that a species is potentially useful in the future does not go extinct [18].

Conservation funds are often disproportionately allocated to a few charismatic animal groups [19]. Using any diversity measure would distribute funds across more species, but conservation of PD specifically aims to spread funds more evenly across the tree of life [20]. For example, priorities based on PD differ from priorities based on the species conservation when species richness and PD hotspots do not have spatial overlap [12,21,22]. This difference is more pronounced if phylogenies have deep radiation events [23].

The use of well-resolved phylogenies in conservation helps minimize taxonomic bias resulting from changing species concepts or geographical differences in naming philosophy or taxonomic effort [24,25]. For example, the same range of morphologic and genetic variation may be known from five species in a well-studied region or a single species in less-studied region. Yet, the area with five species would be much more favoured in a species-based prioritization than prioritization with PD.

The cost–benefit calculation of using PD for conservation is changing given the rapid expansion of spatial and phylogenetic data such as Australia's Virtual Herbarium, and the arrival of global databases such as Timetree (www.timetree.org), the Open Tree of Life (opentreeoflife.org) and the Map of Life (www.mappinglife.org). GIS tools and specialty programs such as Biodiverse [26] help to visualize patterns of diversity across the landscape. Also, the advent of high throughput next generation sequencing techniques has reduced the cost and time in generating large species-level phylogenies [27]. The tools necessary for using PD in conservation are available or becoming available, but a simple framework for integrating PD into a spatial prioritization and a demonstration of how PD might be useful for policy is needed.

## 2. Conservation applications with phylogenies

Perhaps the largest example to date of integrating phylogenies in species conservation is the EDGE list, which prioritizes species for conservation by combining evolutionary distinctiveness (ED) with global endangerment (GE) [8]. ED measures the contribution of each species to the tree of life [8], so is useful for ranking species for conservation such as in setting priorities for which species should be collected and stored in seed banks [28]. However, the actual geographical distributions of species and their co-occurrence are crucial to conservation decisions. Furthermore, priorities change as species or areas become protected or threatened, so complementary-PD measures are a more efficient way of summarizing marginal gains and losses in conservation than scoring approaches [29].

There have been many approaches to combining PD and complementarity to select areas for conservation such as Diversity-PD software [30], greedy algorithms [31] and integer linear programming [32]. Many of these methods are limited to few species or few planning units and do not consider effects across the range of a species (but see Billionnet [33] for a solution that includes dependency in survival probabilities). More recent work has illustrated how phylogenies can be used in a comprehensive planning framework. Strecker [10] used nodes on the phylogeny as conservation units in a spatial prioritization for fishes in the Lower Colorado River Basin in the southwest United States using Zonation software [34].

Here, we aim to enable wider use of PD in conservation by providing a method that links phylogenies, species distribution models (SDMs) and spatial prioritization software. This method could be used for any group of organisms with a phylogeny and distribution data and is especially suited to species that have modelled distributions. Given the recent proliferation of SDMs in the literature and their great potential for use in conservation and management more generally [35], we hope this work will encourage uptake of SDMs for the specific problem of conserving evolutionary diversity. We assign conservation priority with Zonation software, which has the advantage of being a widely used program that can accommodate the complexity of typical conservation problems by including critical factors such as the cost of conservation, species risk status and connectivity between populations across multiple species and large landscapes [36].

We illustrate how this method can be used to quantify current conservation status of evolutionary diversity and to evaluate changes made to a regional conservation policy using a case study of 101 species of eucalypts (*Corymbia* Hill and Johnson, *Angophora* Cav. and *Eucalyptus* L'Hérit, Myrtaceae) in Victoria, Australia. Eucalypts dominate the canopy in nearly every woody vegetation type in Victoria—from shrubs less than 2 m tall to wet forests of *Eucalyptus regnans*, the tallest flowering plant. Victoria has many diverse bioregions [37], but is also the most cleared state in Australia with rates of habitat deterioration continuing to exceed protection and restoration. Eucalypts in Victoria are an excellent case study not only for their ecosystem dominance but also because suitable genetic data are available, and Victoria has exceptional state-level environmental and plant survey data [38].

We address three regional conservation questions: (i) how much PD is represented in the current protected areas? (ii) how much PD can we gain by expanding the protected areas? and (iii) how might a new tourism development policy in national parks impact protection of eucalypt lineages?

### (a) Delineating and modelling species distributions

Any type of distribution data associated with the tips of a phylogeny can be used in this method. In our example, each tip of the phylogeny represents one species, and each species contains distribution information from SDMs or outlined distributions. If SDMs are used, then predicted probabilities of occurrence (from data with known presences and absences) can be used directly in the analyses rather than using a threshold to transform probabilities to binary presence/absence. If predictions are from presence-only data (e.g. herbarium records), then the output should be scaled based on prevalence if suitable prevalence data exist [39]. Many eucalypts have narrow ranges that are overestimated with standard SDMs. Our goal was to develop conservative estimates of species distributions (i.e. underestimating unknown populations in favour of more accurately identifying populations that are known to be present).

We used a range of modelling methods depending on the extent and prevalence of each species (see the electronic supplementary material, appendix S1 for a species list, distribution type and cross-validated area under the receiver operating characteristic curve (AUC) values). Our decision on the type of model was based on a trade-off between having more reliable probabilities of occurrence (which are important in this case because they are propagated through the phylogeny) and missing known populations (by using the smaller, presence–absence dataset that allows probabilities to be determined). Common species were modelled using boosted regression trees (BRTs) [40] with a quadrats dataset from Victoria's Biodiversity Atlas (VBA) accessed October 2013. For range-restricted species, we added the additional records from VBA and the Australia's Virtual Herbarium (AVH) and used MaxEnt [41] for modelling. For the AVH dataset, we removed any records outside of the species natural populations and retained only post-1950 records, because the older records had high spatial uncertainty. However, we may have missed some locations where the species no longer occurs by eliminating these records. Both datasets were clipped to the state of Victoria with a 100 km buffer to limit edge-effects. The final number of unique species × site combinations was 9137 AVH records and 89 454 VBA records. Numbers of records per species ranged from five to over 7000 in the case of wide-ranging *Eucalyptus obliqua*.

For MaxEnt models, we masked out areas beyond the known extent of each species, set a background of the locations of all records of all species and filtered records to exclude duplicate species × locations within 100 m for species with fewer than 200 records and 5 km for species with more than 200 records. We used only hinge features, because they provide smoother response curves, and scaled the output to match prevalence calculated from the VBA quadrats dataset [42]. Ten per cent of data was withheld for model testing and the final models were run on the full datasets. The average 10-fold cross-validated AUC value for withheld data across species was 0.96 (ranging from 0.85 to 0.99) for BRTs and 0.98 (from 0.97 to 1.0) for MaxEnt models. All modelling was done in the R package ‘dismo’ v. 0.8-17 [43] using a set of climatic and edaphic variables described in the electronic supplementary material, appendix S2. Distributions were predicted to 225 m grid cells across the state plus buffer zone including areas that are not currently native woody vegetation. This provided an estimate of how much of the distribution of each species may have been lost owing to past clearing. Modelled distributions are based on relatively recent point data, so the amount of distributions lost is probably underestimated, but nonetheless provides important information about species threat. For additional details on distribution modelling, see the electronic supplementary material, appendix S2.

Distributions of three species that were isolated to a few populations (under 70 records) were delineated in ArcMap v. 10.2 based on species descriptions and expert knowledge. Polygons were assigned probabilities based on expert opinion and/or species descriptions.

### (b) Phylogeny

We assembled a sequence matrix of 96 species plus outgroup taxa based on four markers, two nuclear ITS, ETS, and two nuclear *matK* and the *psbA-trnH* intergenic spacer. Sequences were sourced from the alignment prepared for a larger eucalypt phylogeny [44]. A Bayesian analysis was performed using MrBayes v. 3.2 compiled on the CSIRO Burnett supercomputer cluster. The Monte Carlo Markov chain was run for 40 × 10^6^ generations and convergence was achieved with a final split frequency value of 0.041825. The final tree was exported as a nexus file. Five species with missing molecular data were inserted into the nexus phylogeny file at the stem node shared with assumed most closely related species as in Rosauer *et al.* [45] with a branch length of zero (see the electronic supplementary material, appendix S1). The Victorian eucalypt phylogeny is shown in electronic supplementary material, appendix S3. Relationships between major groups (genera and subgenera) are in agreement with existing eucalypt phylogenies [46,47].

### (c) Linking species distribution models to the phylogeny

**Figure 1.**
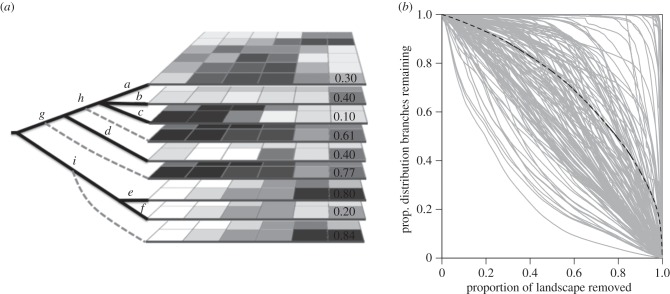
Each branch on the phylogeny is considered a conservation ‘unit’ in the spatial prioritization and has an associated raster grid with a probability of occurrence in each cell (illustrated in lower right cell in *a*). Rasters for tip branches (i.e. species) are estimated with SDMs and rasters for internal branches (dotted lines) are calculated according to equation (2.1). (*b*) During the prioritization process, as cells are removed from the landscape, the proportion of the original distribution of each branch that is still remaining is recorded (grey solid lines, black dashed line shows the average across all branches). See text for further details on the prioritization process.

Each cell in a grid has a modelled probability of occurrence (from SDMs) for each species (figure 1*a*). Each species is a terminal branch of the phylogeny. We calculated the probability of occurrence for each internal branch in each cell. An internal branch occurs if any of the descendent species occur in that cell. Thus,2.1
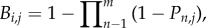
where *B_i_*_,*j*_ is the probability of an internal branch (*i*) occurring in cell *j*, *m* is the number of descendent species downstream of this internal branch and *P_n_*_,*j*_ is the probability of descendent species *n* occurring in cell *j*.

Probabilities of occurrences for branch lengths were calculated in R [48]. Owing to the large size of the raster files (millions of pixels), calculation of probability layers for internal branches was performed directly in raster format using a combination of customized functions and functions available within the ‘raster’ package [43]. Attributes of the phylogeny were extracted using functions from the ‘ape’ package [49].

### (d) Spatial prioritization

We used Zonation v. 4.0 for the spatial prioritization. Zonation produces a conservation priority of sites (or grid cells) in a given landscape based on representation of biodiversity features (e.g. species or, as in this case, branches), feature weights and the cost of protecting a site. It starts by assuming that everything in the landscape is protected and then iteratively removes grid cells with the least conservation benefit (i.e. the least marginal loss) [36].

At each step, the remaining proportion of the distribution of each feature is calculated to determine which cell is the least valuable based on principles of complementarity and irreplaceability, and hence will be removed next (figure 1*b*). Each time Zonation recalculates the proportion of the distribution of each branch remaining, it uses the probabilities in each cell that were previously calculated according to equation (2.1). This means that even though branches are independent units, the branches remain mathematically linked in the phylogenetic hierarchy at each step. We used both the basic Core Area Zonation (CAZ), which removes cells based on the maximum value in a cell for any given feature, and the Additive Benefit Function (ABF), which sums values in cells [34]. The ABF approach represents total PD slightly better, but, as in other cases, the distribution of individual biodiversity features (phylogenetic branches in this case) were preserved better with CAZ than with ABF [36], so we present the results of CAZ here.

The performance of a Zonation solution is typically measured by how original distributions of features are retained by sites that correspond to a specific fraction of the entire landscape, e.g. the best 10% of total area [36]. Here, instead of individual branches, we are evaluating spatial prioritizations based on how well they represent total PD. Therefore, we calculated the proportion of PD remaining in the landscape at each step in the cell removal according to2.2
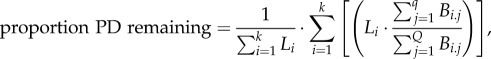
where *k* is branches on the phylogeny, *q* is the remaining cells of native woody vegetation on the landscape, *Q* is the initial number of cells (all cells present), *B_i_*_,*j*_ is the probability of occurrence of branch *i* in cell *j* and *L* is the length of branch *i*. With all currently existing native woody vegetation represented as cells on the landscape, the entirety of each branch is represented and PD is the sum of all branch lengths as in Faith [5]. As grid cells are removed, loss of branches is represented by the proportion of the spatial distribution of each branch remaining weighted by branch length.

We ran Zonation for various scenarios (table 1) using mask files which alter the cell removal order to either force in or force out areas from the top priorities, such as existing protected areas or proposed development areas. The impact of existing and proposed land use types can then be quantified by comparing the results of an altered solution to an unconstrained optimal solution [50]. We use the proportion of PD remaining (equation (2.2)) to evaluate scenarios of reserve expansion and contraction. We ran the different prioritization scenarios at the resolution of the modelled distributions (225 m resolution, 4 481 600 cells) across the state of Victoria with the warp factor (number of cells removed at a time) set at 100 and without considering connectivity. Portions of the ranges of species and clades that have already been lost to clearing was considered by first ranking the cleared areas (some of which have modelled species ranges), then ranking all areas that are currently native woody vegetation (table 1). Including cleared land in the prioritization integrates the proportion of the spatial distribution of species that have already been cleared.

**Table 1.** List and description of Zonation runs. (The optimal prioritization can be altered using mask files, which tell the program that some areas have predefined hierarchy, and removes categories of grid cells in specified order.)

run	mask files	description
optimal solution	none	prioritize all of Victoria
optimal solution for remaining native woody vegetation	0—not native woody vegetation 1—native woody vegetation	prioritize remaining native woody vegetation in Victoria
evaluate conservation reserves	0—not native woody vegetation 1—native woody vegetation outside current conservation areas 2—native woody vegetation inside current conservation areas	prioritize areas inside and outside conservation reserves to determine how much PD is already protected and how much remains outside the current extent of the reserve systems
scenario 1: expand conservation reserves	0—not native woody vegetation 1—native woody vegetation outside current conservation areas 2—native woody vegetation inside current conservation areas	compare amount of PD protected in reserves with that protected if the area of conservation reserves was increased by 5 or 20%
scenario 2: tourism development in national parks	0—outside native woody vegetation 1—native woody vegetation outside national parks 2—native woody vegetation in national parks and open for development 3—native woody vegetation in national parks and protected from development	document the distribution of PD within national parks available for tourism and map vulnerable areas

## 3. Ranking the landscape for phylogenetic diversity

**Figure 2.**
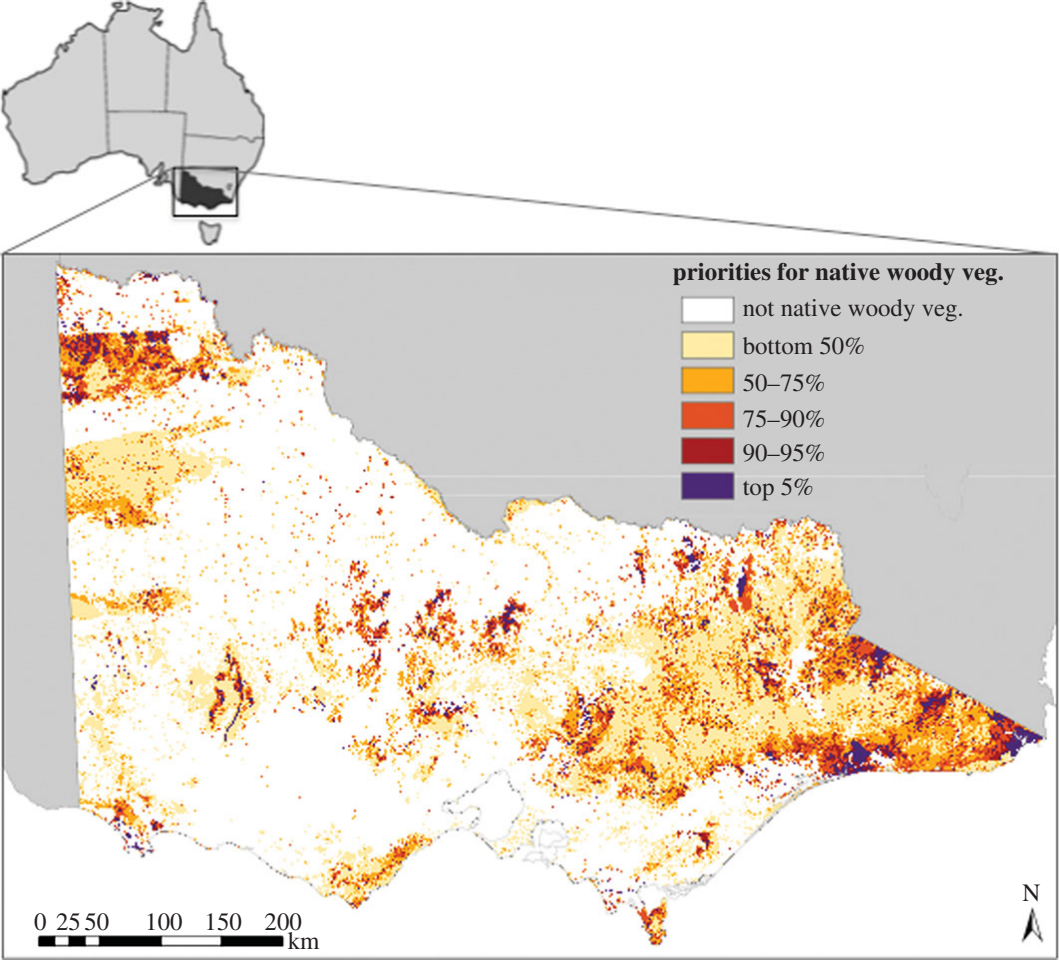
Priority ranking for all native woody vegetation in Victoria (inset), Australia. Ranks are the conservation benefit based on the PD of eucalypts not considering land tenure.

The most valuable areas for conservation of PD are distributed throughout the state. Notable regions include the mallee eucalypts in Murray-Sunset National Park in the northwest, the Grampians National Park in the west, the heavily degraded box-ironbark forests in central Victoria and the East Gippsland region in the eastern part of the state (figure 2). This map shows the relative conservation importance of areas across Victoria for PD, ignoring any existing land tenure. In order to identify next conservation priorities in a cost-effective manner, one needs to take into account that some species are already protected by existing reserve network. For example, the northwest part of the state is an important resource for PD, but nearly all of the remaining native vegetation is already protected within Murray-Sunset National Park.

## 4. How well is phylogenetic diversity represented in national parks?

**Figure 3.**
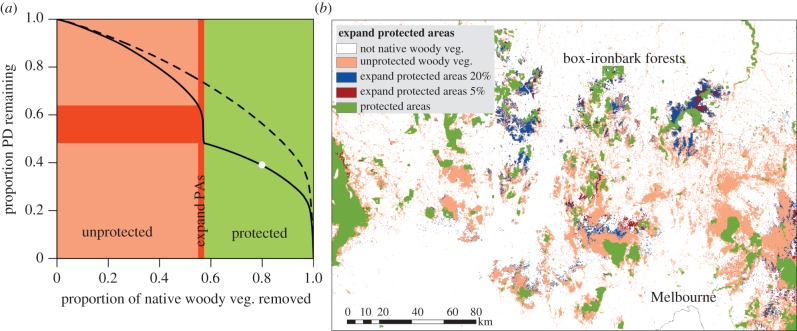
Hypothetical protected area expansion for Victoria as PD-loss curves (*a*) and a map showing these priority areas for a subset of the state (*b*). Loss of PD is represented as the proportion of PD lost as cells of native woody vegetation are removed. Colours represent the protection status of native woody vegetation (pink, unprotected; green, protected; red, expand protected areas by 5%; and blue, expand by 20%). (*a*) The dashed black line is the amount of PD that could be retained if all areas of native woody vegetation were available for protection. The solid black line is the amount of PD captured by the actual layout of protected areas (and also shows how much PD could be added with protected area expansion). The *y*-axis difference between the dashed and solid lines is the difference between the actual amount of PD that is protected and the amount that could be protected by a given proportion of land if protected areas were expanded efficiently. Red blocks are the amount of PD (horizontal block) and land area (vertical block) that could be included in protected areas if they were expanded by 5% of their existing land area. See the electronic supplementary material, appendix S4 for a statewide map.

Widespread clearing has left less than 40% of Victoria with native woody vegetation. Of that remaining vegetation, 43% is protected in nature reserves, most of which are national parks. The current configuration of conservation reserves is not optimal—only 48% of the total PD is currently located in protected areas compared with 74% protected if reserves were located optimally following the prioritization in figure 2 (dashed and solid lines in figure 3*a*).

If the protected areas were to be expanded in a cost-efficient manner by 5% (less than 1% of the area of the state), an additional 33% of PD could be protected (totalling 64% of the PD remaining today as native woody vegetation; red in figure 3*a*). The hypothetical protected area expansion is concentrated in central Victoria (figure 3*b*) and the South East Corner Bioregion (electronic supplementary material, figure S4), but there are many smaller locations throughout the state. Various other sizes and configurations of reserve expansions could be considered. In a less realistic scenario, we could increase the protected PD of eucalypts by 50% with a 21% expansion of protected areas (figure 3*b*).

## 5. Evaluating a policy change to the protected area system

National parks are an important repository of eucalypt PD but many areas within national parks are not fully protected because they are now available for tourism development. In 2013, portions of the national park system in Victoria were made available for tourism development under the ‘Tourism Investment Opportunities of Significance’. Development must be sensitive to the park values, environmentally sustainable and must be a net public benefit, which includes increasing public access to park resources [51].

**Figure 4.**
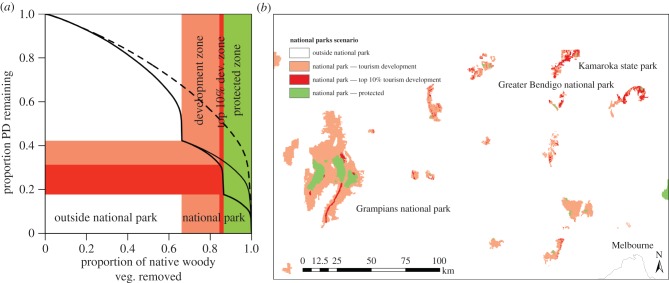
Tourism development in national parks as PD-loss curves (*a*) and map (*b*). (*a*) The dashed line is the optimal solution for all woody native vegetation (as in figure 3*a*). The thin black line represents PD retained in the optimal configuration of protected zones within national parks. The thick black line is the PD retained when national park tourism development zones (pink/red blocks) are prioritized first, followed by protected zones in national parks (green block). The top 10% of tourism development zones ranked by PD contribution are shown as red on the graph (*a*) and the map (*b*). These development zones could be transferred to protected zones to capture 72% more PD than currently is found in protected zones. The map shows one region north and west of the major metropolitan area, Melbourne, which contains many valuable national park lands open for tourism development (see the electronic supplementary material figure S5 for the statewide map).

We refer to areas within national parks as ‘protected zones' and ‘development zones' depending on whether they are open for tourism development or not. National parks contain 42% of the PD of native woody vegetation, over half of which is found in development zones (figure 4). If the development zones were re-distributed to avoid as much eucalypt PD as possible, nearly twice as much PD could be represented in the protected zones (33% of the PD rather than the current 18%; figure 4*a*).

Transferring even 10% of the area of development zones to protected zones would increase the amount of PD that is fully protected to 31% (figure 4*a*). Many of these valuable PD resources within development zones (red in figure 4*b*) are located in parks that are easily accessible from the metropolitan city of Melbourne and are, therefore, potentially at high risk of being developed for tourism. Extending the protection zone to the areas in red would help ensure that important evolutionary diversity is fully protected.

**Figure 5.**
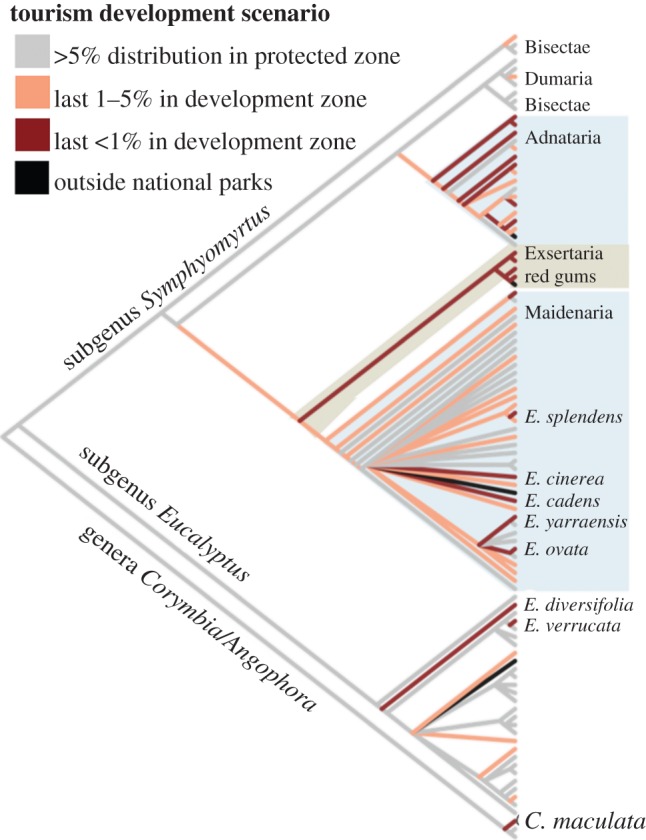
Parts of the phylogeny vulnerable from tourism development in national parks. Four species are found entirely outside of national parks (black branches). Grey bars indicate when greater than 5% of the original spatial distribution of a branch is found on protected zones within national parks. Branches that are pink and red have 1–5% or less than 1% of their respective distributions in protected zones within national parks.

We can also visualize which branches on the phylogeny may be vulnerable to tourism development (figure 5). For this we consider the entire spatial distribution of each branch, including the portions of the distribution that have already been cleared. We calculate the proportion of the distribution of each branch that is located outside national parks and on protected and development zones in national parks. Potentially affected species are clustered on the phylogeny with subgenera *Symphyomyrtus* being particularly vulnerable (figure 5). The distributions of 20 species fall below 1% of their original distribution meaning that the last protected portion of their distribution is in the tourism development zone. The last remaining 1% of the entire red gum clade (including river red gum, *Eucalyptus camaldulensis*) is located in tourism development areas. Many red gums, such as *E. camaldulensis* are widespread and, therefore, might not be impacted by any actions in national parks. However, it is notable that the entire clade is under-represented with all branches having less than 1% of their distributions found in protected zones within national parks (figure 5). Some other species are located almost exclusively in tourism development zones. For example, nearly the entire range of Serra Range gum (*Eucalyptus verrucata*) is located in a tourism development zone. Many more species and the entire Adnataria clade fall below 5% remaining in protected zones (figure 5)

It is important to note that the tourism development zones will not be fully developed, and therefore, not all of the PD located in these zones will be threatened or lost. However, one of the requirements of any development is that it has to be a net public benefit, and increasing visitor access is considered a benefit [51], so impact could potentially extend beyond the actual development. This analysis has shown that some areas within development zones are particularly important pools of PD. Development zones contain a number of species and one entire clade that are unrepresented in national parks or under-represented in the protected zones within them. Less than 20% of the PD remaining as native vegetation is located on protected zones within national parks. Small but strategically located expansion of protected zones within national parks could increase protection of species and lineages.

## 6. Other considerations when using phylogenetic diversity in spatial prioritization

Phylogenies are hypotheses with uncertainty arising from many sources including the underlying model of evolution, which may affect conservation predictions based on them [25]. Eucalypts are a particularly challenging taxonomic group, which sometimes do not fully confirm to a bifurcating tree in cases of hybridization and introgression [52,53] and parallel evolution [54]. Fine-scale phylogenetic relationships will be increasingly understood as new molecular technologies emerge [46,55]. In spatial prioritization with Zonation, spatial uncertainty can be directly incorporated into the prioritization [56]. However, much of the phylogenetic uncertainly involves the tree topology, and changing the topology changes the conservation features. One way to account for phylogenetic uncertainty is to run the prioritization multiple times with different estimates of the phylogeny to obtain a distribution around estimates. A similar type of uncertainty analysis could be done in Zonation, but is beyond the scope of this paper.

Another consideration for any spatial prioritization is the effect of bounding the study area, because priorities tend to be inflated near boundaries that bisect species distributions [57]. Further research is needed to understand boundary effects for PD specifically. Boundaries might be an issue for PD even if all species are found entirely within the study area, because the range of internal branches might be underestimated if related species occur elsewhere. In this case, we suspect inflated priorities in the east and northwest, where some branches extend into New South Wales or South Australia. Bounding the study area at Victoria is justified if the aim of the study is to manage Victoria's resources, reflecting its separate laws and regulations from surrounding states, and making use of the state's independent datasets. Other options would be to weight endemic branches higher than branches that extend beyond the study area. For example, the *Corymbia* clade, which is rare in Victoria but widespread elsewhere, could be given a lower priority. One of the benefits of using Zonation or a similar software is that any species (or branch) could be weighted for any desirable attribute such as threat categories or functional attributes. However, if threat is included, such as International Union of Conservation of Nature threat status, it is important to keep in mind that Zonation considers rarity by the proportion of the distribution of a species remaining, so weighting by threat status may over-emphasize listed species in the prioritization.

## 7. Policy recommendations and conservation applications

Our analysis suggests that the protected zones within national parks could be modestly extended to include the most valuable 10% of the tourism development zones for eucalypt diversity in Victoria. The expansion of the protected zones would reduce chances that species or even clades are negatively impacted. Given that eucalypts provide the forest habitat for many species, areas important for eucalypt diversity may also contain high diversity for other organisms, but similar analyses could be done for other groups to determine additional diverse and threatened locations.

Given the multitude of concerns facing policy-makers and managers, finding overlap between areas that contain valuable evolutionary diversity and areas important for other concerns may increase the likelihood of PD being considered. The box-ironbark forests in central Victoria (Victorian Midlands IBRA Bioregion and Goldfields Sub-bioregion) are one good example of a region designated high priority in our analyses and other conservation rankings, such as Trust for Nature spatial prioritization [58]. In our analysis, parts of the box-ironbark region were ranked highly across all native vegetation, were included in reserve expansion scenarios, and were in the highest 10% of the national parks areas open for tourism development. Edge-effects would have a minimal influence as the box-ironbark region is centrally located. The box-ironbark region is heavily degraded from the 1850s gold rush, logging, agriculture, development and aridification from climate change [59]. Eucalypts provide critical habitat for numerous organisms, especially nectar-eating birds which depend on year-round flowering by different eucalypts [60]. The box-ironbarks should be reinforced as a high priority because, in addition to having many threatened species, they also are an important resource for preserving eucalypt evolutionary history.

## 8. Conclusion

Real-world conservation efforts that consider PD are lagging behind interest from the scientific community. Here, we attempted to facilitate the use of PD in conservation by providing user-friendly methods and demonstrating how PD can be relevant for conservation decisions. This method links two rapidly expanding data sources—phylogenies and SDMs—with widely used spatial prioritization software. PD can be used in hypothetical or actual protected area scenarios for any study group that has a phylogeny and distribution data. For eucalypt trees in Victoria, a small 5% expansion to protected areas (less than 1% of the state), could capture 33% more PD. Following a recent policy change opening national parks to development, only 11% of PD is fully protected in Victoria, with some clades particularly vulnerable. However, small changes to development zones could greatly improve the outlook for species and lineages. This framework enables PD to be included with other economic, ecological or sociological factors that are needed in complex real-world planning.

## Supplementary Material

Supplementary material
