# Supplementary material for: Phylogenetic diversity meets conservation policy: small areas are key to preserving eucalypt lineages
Source: Philos Trans R Soc Lond B Biol Sci. 2015 Feb 19;370(1662):20140007. doi: 10.1098/rstb.2014.0007 (PMC4290421; doi:10.1098/rstb.2014.0007)

Pollock, L.J. *et al.* 2015 Phylogenetic diversity meets conservation policy: small areas are key to preserving eucalypt lineages. *Phil. Trans. R. Soc. B* **370** doi: 10.1098/rstb.2014.0007

Supplementary materials

Appendix S1: Eucalypt species list

The species, type of distribution, prevalence- the total probability of occurrence of each species across all cells in the landscape (‘Sum Occur.’), cross-validated Area Under the ROC Curve (AUC) statistic, common name, molecular sample ID and naming authority. The distribution of a species was delineated in ArcMap or modelled using Boosted Regression Trees (BRTs) or MaxEnt (with or without a mask bounding the distribution). Cross-validated AUC values are from the 10% of data withheld from the model for each of 10 replicates (final models were fit with all data) for BRT models and MaxEnt models (in brackets). Five species were missing molecular sequence information. The five missing species were placed in the phylogeny in the clade of the closely related species with a branch length of zero.

Eucalypts are a challenging taxonomic group, and a variety of sources were used to make decisions regarding species names and distributions including EUCLID version 3, Nicolle (2006) and expert opinion. This list does not include subspecies and some recently described species in Victoria including *Eucalyptus macmahonii*, *molyneuxxii*, *Eucalyptus silvestris* (included with *E. microcarpa*), *Eucalyptus wimmerensis* (included as *E. viridis*).

Species	Distribution Type	cvAUC	Sum Occur.	Common Name	Sample ID	Authority
Angophora floribunda	MaxEnt-Mask	(1.000 ± 0.000)	669	Apple	LAN107	(Sm.) Sweet
Corymbia gummifera	MaxEnt-Mask	(0.999 ± 0.001)	815	Red bloodwood	680	(Sol. ex Gaertn.) K.D.Hill & L.A.S.Johnson
Corymbia maculata	MaxEnt-Mask	(0.999 ± 0.001)	777	Spotted gum	ANBG7800470	(Hook.) K.D.Hill & L.A.S.Johnson
Eucalyptus agglomerata	BRTs	0.985 ± 0.011	2334	Blue-leaf Stringybark	673	Maiden
Eucalyptus aggregata	MaxEnt-Mask	(0.999 ± 0.001)	609	Black Gum	580	H. Deane & Maiden
Eucalyptus alaticaulis	BRTs	0.991 ± 0.011	6312	Grampians Grey-gum	371	R.J. Watson & Ladiges
Eucalyptus albens	BRTs	0.991 ± 0.006	40996	White Box	661	Benth.
Eucalyptus alligatrix	MaxEnt	0.991 ± 0.012 (0.994 ± 0.003)	12164	Silver Stringybark	167	L.A.S. Johnson & K.D. Hill
Eucalyptus angophoroides	BRTs	0.981 ± 0.030	2277	Apple Box	158	R.T. Baker
Eucalyptus arenacea	BRTs	0.978 ± 0.043	28817	Desert Stringybark	MC10652	Marginson & Ladiges
Eucalyptus aromaphloia	BRTs	0.962 ± 0.031	35849	Scentbark	CANB564611	L.D. Pryor & J.H. Willis
Eucalyptus baueriana	BRTs	0.976 ± 0.044	7648	Blue Box	160	Schauer
Eucalyptus baxteri	BRTs	0.944 ± 0.009	121553	Brown Stringybark	8701806	(Benth.) Maiden & Blakely ex J.M. Black
Eucalyptus behriana	BRTs	0.984 ± 0.007	100531	Bull Mallee	(populnea)	F. Muell.
Eucalyptus blakelyi	BRTs	0.992 ± 0.008	65632	Blakely's Red-gum	211	Maiden
Eucalyptus bosistoana	BRTs	0.982 ± 0.018	5329	Coast Grey-box	671	F. Muell.
Eucalyptus botryoides	BRTs	0.968 ± 0.015	35070	Southern Mahogany	304	Sm.
Eucalyptus bridgesiana	BRTs	0.942 ± 0.012	51892	But But	257	R.T. Baker
Eucalyptus brookeriana	MaxEnt	0.903 ± 0.154 (0.983 ± 0.007)	15753	Brooker's Gum	617	A.M. Gray
Eucalyptus cadens	MaxEnt-Mask	(0.999 ± 0.001)	899	Warby Range Swamp-gum	617	J.D. Briggs & Crisp
Eucalyptus calycogona	BRTs	0.988 ± 0.004	98412	Red Mallee	362	Turcz.

Eucalyptus camaldulensis	BRTs	0.984 ± 0.002	637495	River Red-gum	ANBG8401853	Dehnh.
Eucalyptus camphora	BRTs	0.968 ± 0.017	40476	Mountain Swamp-gum	621	L.A.S. Johnson & K.D. Hill
Eucalyptus cephalocarpa	BRTs	0.956 ± 0.008	20416	Mealy Stringybark	178	Blakely
Eucalyptus chapmaniana	BRTs	0.966 ± 0.053	12476	Bogong Gum	(rubidaANBG732 895)	Cameron
Eucalyptus cinerea	MaxEnt-Mask	(1.000 ± 0.000)	591	Mealy stringybark	8703938	F.Muell. ex Benth.
Eucalyptus consideniana	BRTs	0.961 ± 0.011	45139	Yertchuk	219	Maiden
Eucalyptus conspicua	BRTs	0.944 ± 0.094	2077	Silver Swamp Stringybark	113	L.A.S. Johnson & K.D. Hill
Eucalyptus costata	BRTs	0.992 ± 0.002	211175	Yellow Mallee	283	F. Muell. & Behr ex. F. Muell.
Eucalyptus crenulata	MaxEnt	(0.999 ± 0.000)	18218	Buxton Gum	307	Blakely & Beuzev.
Eucalyptus croajingolensis	BRTs	0.950 ± 0.008	22527	Gippsland Peppermint	156	L.A.S. Johnson & K.D. Hill
Eucalyptus cyanophylla	MaxEnt	0.998 ± 0.002 (0.999 ± 0.000)	14808	Blue-leaf Mallee	338	Brooker
Eucalyptus cypellocarpa	BRTs	0.923 ± 0.005	209873	Mountain Grey-gum	665	L.A.S. Johnson
Eucalyptus dalrympleana	BRTs	0.985 ± 0.004	38210	Mountain Gum	ANBG7804378	Maiden
Eucalyptus delegatensis	BRTs	0.991 ± 0.002	74188	Alpine Ash	CANB499445	R.T. Baker
Eucalyptus denticulata	BRTs	0.992 ± 0.006	6948	Errinundra Shining Gum	378	I.O. Cook & Ladiges
Eucalyptus diversifolia	MaxEnt-Mask	(1.000 ± 0.000)	302	Coast Gum	390	I.J. Wright & Ladiges
Eucalyptus dives	BRTs	0.941 ± 0.010	232441	Broad-leaf Peppermint	ANBG7809979	Schauer
Eucalyptus dumosa	BRTs	0.987 ± 0.002	391068	Dumosa Mallee	329	A. Cunn. ex J. Oxley
Eucalyptus dwyeri	MaxEnt-Mask	(0.998 ± 0.000)	329	Dwyer's red gum	9406593	Maiden & Blakely
Eucalyptus elaeophloia	MaxEnt	(1.000 ± 0.000)	1706	Olive Mallee	372	Chappill, Crisp & Prober
Eucalyptus elata	BRTs	0.961 ± 0.006	20083	River Peppermint	ANBG73059	Dehnh.
Eucalyptus eugenioides	Delineated		52	Thin-leaved Stringybark	578	Sieber ex Spreng.
Eucalyptus fasciculosa	MaxEnt-Mask	(1.000 ± 0.000)	285	Pink Gum	289	F. Muell.
Eucalyptus fastigata	BRTs	0.986 ± 0.003	20852	Cut-tail	8603812	H. Deane & Maiden
Eucalyptus fraxinoides	MaxEnt-Mask	(0.994 ± 0.001)	2266	White Ash	751650	H. Deane & Maiden

Eucalyptus froggattii	MaxEnt	(0.991 ± 0.004)	66689	Kamarooka Mallee	479	Blakely
Eucalyptus fulgens	BRTs	0.964 ± 0.027	5603	Green Scentbark	9707943	Rule
Eucalyptus glaucescens	BRTs	0.930 ± 0.134	1848	Tingaringy Gum	218	Maiden & Blakely
Eucalyptus globoidea	BRTs	0.957 ± 0.006	130757	White Stringybark	729	Blakely
Eucalyptus globulus	BRTs	0.925 ± 0.014	102151	Southern Blue-gum	ANBG85001816	Labill.
Eucalyptus goniocalyx	BRTs	0.959 ± 0.008	202510	Bundy	669	F. Muell. ex Miq.
Eucalyptus gracilis	BRTs	0.987 ± 0.008	135366	Yorrell	202	F. Muell.
Eucalyptus ignorabilis	BRTs	0.848 ± 0.173	2558	Grey Scentbark	379	L.A.S. Johnson & K.D. Hill
Eucalyptus kitsoniana	MaxEnt	0.818 ± 0.353 (0.971 ± 0.021)	57491	Bog Gum	110	Maiden
Eucalyptus kybeanensis	MaxEnt	(0.983 ± 0.011)	13622	Mallee Ash	AHTE19	Maiden & Cambage
Eucalyptus largiflorens	BRTs	0.992 ± 0.001	288327	Black Box	CANB785604	F. Muell.
Eucalyptus leptophylla	BRTs	0.989 ± 0.005	205763	Slender-leaf Mallee	200	F. Muell. ex Miq.
Eucalyptus leucoxylon	BRTs	0.973 ± 0.011	258249	Yellow Gum	716	F. Muell.
Eucalyptus litoralis	BRTs	0.996 ± 0.009	733	Otway Grey-gum	(cypellocarpa665)	Rule
Eucalyptus mackintii	MaxEnt	(0.966 ± 0.019)	23137	Gippsland Stringybark	308	Kottek
Eucalyptus macrorhyncha	BRTs	0.956 ± 0.004	292817	Red Stringybark	761	F. Muell. ex Benth.
Eucalyptus mannifera	BRTs	0.970 ± 0.017	71139	Brittle Gum	610	Mudie
Eucalyptus melliodora	BRTs	0.948 ± 0.006	218464	Yellow Box	ANBG662272	A. Cunn. ex Schauer
Eucalyptus microcarpa	BRTs	0.986 ± 0.004	383519	Grey Box	475	(Maiden) Maiden
Eucalyptus mitchelliana	MaxEnt-Mask	(1.000 ± 0.000)	74	Buffalo Sallee	635	Cambage
Eucalyptus muelleriana	BRTs	0.958 ± 0.010	48398	Yellow Stringybark	159	A.W. Howitt
Eucalyptus neglecta	MaxEnt	(0.987 ± 0.006)	77213	Omeo Gum	72027	Maiden
Eucalyptus nitens	BRTs	0.985 ± 0.011	7700	Shining Gum	ANBG651034	(H. Deane & Maiden) Maiden
Eucalyptus nortonii	BRTs	0.981 ± 0.024	3465	Silver Bundy	664	(Blakely) L.A.S. Johnson
Eucalyptus obliqua	BRTs	0.895 ± 0.006	458305	Messmate Stringybark	RoyalCanberraGo lfAHT21	L'Her.

Eucalyptus odorata	MaxEnt		18763	Peppermint Box	(viridis767)	Behr in D.F.L. von Schlechtendal
Eucalyptus oleosa	BRTs	0.993 ± 0.002	306000	Oil Mallee	58	F. Muell. ex Miq.
Eucalyptus ovata	BRTs	0.914 ± 0.010	291629	Swamp Gum	ANBG475702	Labill.
Eucalyptus pauciflora	BRTs	0.986 ± 0.005	92261	Snow Gum	ANBG9303293	Sieber ex Spreng.
Eucalyptus perriniana	MaxEnt	0.957 ± 0.086 (0.988 ± 0.007)	20150	Spinning Gum	742	F. Muell. ex Rodway
Eucalyptus phenax	MaxEnt	(0.988 ± 0.005)	167325	Green-leaf Mallee	375	Brooker & Slee
Eucalyptus polyanthemos	BRTs	0.962 ± 0.006	221409	Red Box	659	Schauer
Eucalyptus polybractea	BRTs	0.981 ± 0.048	6894	Blue Mallee	760	R.T. Baker
Eucalyptus porosa	MaxEnt	0.944 ± 0.106 (0.987 ± 0.004)	194668	Black Mallee-box	MC10664	F. Muell. ex Miq.
Eucalyptus radiata	BRTs	0.901 ± 0.012	369529	Narrow-leaf Peppermint	732	Â Sieber ex DC.
Eucalyptus regnans	BRTs	0.988 ± 0.003	66514	Mountain Ash	ANBG8501815	F. Muell.
Eucalyptus rubida	BRTs	0.949 ± 0.007	125974	Candlebark	ANBG732895	H. Deane & Maiden
Eucalyptus sabulosa	BRTs	0.975 ± 0.043	11253	Wimmera Scentbark	CANB564611	Rule
Eucalyptus saxatilis	MaxEnt-Mask	(0.998 ± 0.001)	365	Rock Mallee	7906246	J.B. Kirkp. & Brooker
Eucalyptus serraensis	MaxEnt-Mask	(0.996 ± 0.001)	149	Grampians Stringybark	MC10646	Ladiges & Whiffin
Eucalyptus sideroxylon	BRTs	0.996 ± 0.008	7524	Mugga	750609	A. Cunn. ex Woolls
Eucalyptus sieberi	BRTs	0.963 ± 0.003	119720	Silvertop Ash	ANBG8411661	L.A.S. Johnson
Eucalyptus smithii	BRTs	0.955 ± 0.014	11689	Gully Gum	217	R.T. Baker
Eucalyptus socialis	BRTs	0.988 ± 0.001	163111	Grey Mallee	276	F. Muell. ex Miq.
Eucalyptus splendens	MaxEnt-Mask	(1.000 ± 0.000)	434	Apple Jack	303	Rule
Eucalyptus stellulata	BRTs	0.977 ± 0.009	11917	Black Sallee	9102880	Sieber ex DC.
Eucalyptus strzeleckii	MaxEnt	0.871 ± 0.168 (0.997 ± 0.001)	44555	Strzelecki Gum	(brookeriana617)	Rule
Eucalyptus tereticornis	BRTs	0.994 ± 0.010	30890	Gippsland Red-gum	769	Brooker & Slee
Eucalyptus tricarpa	BRTs	0.969 ± 0.005	76694	Red Ironbark	8701791	(L.A.S. Johnson) L.A.S Johnson & KD Hill
Eucalyptus verrucata	Delineated		8	Mt Abrupt Stringybark	715	Ladiges & Whiffin

Eucalyptus victoriana	Delineated		65	Victoria Range Stringybark	712	Ladiges & Whiffin
Eucalyptus viminalis	BRTs	0.881 ± 0.006	340258	Manna Gum	615	Labill.
Eucalyptus viridis	BRTs	0.993 ± 0.007	19847	Green Mallee	767	R.T. Baker
Eucalyptus willisii	BRTs	0.974 ± 0.011	32636	Promontory Peppermint	CANB468525	Ladiges, Humphries & Brooker
Eucalyptus yarraensis	MaxEnt	0.887 ± 0.092	161050	Yarra Gum	8005851	Maiden & Cambage

References:

Nicolle, D. 2006. Eucalypts of Victoria and Tasmania. Blooming Books, Melbourne, Australia

EUCLID software Version 3. 2006. Centre for Plant Biodiversity Research. Australian National Herbarium, Canberra, Australia

Appendix S2: Supplemental Methods

Species Distribution Modelling:

Species distributions were modeled using MaxEnt, Boosted Regression Trees (BRTs) or by manually outlining populations of rare species. The decision about which technique to use depended on the spatial extent of the species and available data.

Variables for SDMs included climate variables, edaphic variables, landscape variables, and one variable representing condition of native vegetation (Table S2). Climate variables (summer rainfall and minimum temperature) tended to be the most important across all models (Fig. S2). However, all variables were important in some models. For example, the vertical distance above wetlands was the most important variable in the model for River Red Gum (*E. camaldulensis*). The rare species *E. cadens* (listed as vulnerable federally and within the state) is best described by soil radiometric K (Cook *et al*. 1996), which indicates higher clay content in the soil.

Cross-validated AUC values averaged across species were 0.96 (ranging from 0.85 to 0.99) for BRTs and 0.98 (from 0.97 to 1.0) for MaxEnt models. The spatial overlap and model fit was evaluated using both types of modelling for select species. Model fits were similar between models (within AUC of 0.05) except for rare species that had very few points within the plots dataset used in the BRT modelling. In those cases, AUC values were higher for the MaxEnt models (Cross-validated values for both BRTs and MaxEnt in Appendix S1). Perhaps more importantly, the MaxEnt models also captured more known populations at the edge of the ranges of rare species, because they included occurrence records in addition to the plots datasets.

Range-restricted species were masked (see text) after the species distribution modelling. We delineated the known extent of the species by manually outlining point locations in ArcMap 10.2 and consulting species descriptions when necessary. In addition to the outline of the extent of the population, we also delineated polygons with the distribution that we were more or less certain of species occurrence and assigned probabilities based on expert opinion. In the case of *E. victoriana* and *E. verrucata*, many populations are visible in aerial photography and most were also confirmed on the ground. We converted the polygons to a grid in ArcMap 10.2 that matched the SDM layers.

We then assigned all values from the SDM outputs that fell outside the delineated distributions to zero using the Spatial Analyst toolset in ArcMap 10.2. All final SDM outputs were rounded to four decimal places (0.0001 being the lowest probability allowed) using the Raster package in R. Then, the grid cells were multiplied by four (to reduce file size) and wrote to an ascii file.

Table S2. List and description of spatial data used in species distribution models (SDMs) and categories for spatial prioritization.

Modelling variables	Description
Wetness Index	Topographic Wetness Index a compound terrain attribute (sensu Bevan and Kirby 1979) implemented using the Shuttle Radar Topography Mission (SRTM) Digital Elevation Model and TOPOCROP Version 2.1 (Schmidt 2002)
Potassium	Radiometric Data – Radioelement count of Potassium. Sourced from Various Federal and State Agencies.
Thorium	Radiometric Data – Radioelement count of Thorium. Sourced from various Federal and State Agencies.
Summer Max Temp	Mean maximum temperature January developed with ANUCLIM (Houlder et al 2000) applied to 30 m Shuttle Radar Topography Mission (SRTM) Digital Elevation Model.
Winter Min Temp	Mean minimum temperature July developed with ANUCLIM (Houlder et al 2000) applied to 30 m Shuttle Radar Topography Mission (SRTM) Digital Elevation Model.
Summer Rainfall	Mean Rainfall in January developed with ANUCLIM (Houlder et al 2000) applied to 30 m Shuttle Radar Topography Mission (SRTM) Digital Elevation Model.
Winter Rainfall	Mean Rainfall July developed with ANUCLIM (Houlder et al 2000) applied to 30 m Shuttle Radar Topography Mission (SRTM) Digital Elevation Model.
Distance above wetlands	Log transformed vertical distance above mapped water features - wetlands and waterbodies (Conrad 2002). Implemented on 30 m Shuttle Radar Topography Mission (SRTM) Digital Elevation Model.
Dist. above major streams	Log transformed vertical distance major rivers (Conrad 2002). Implemented on 30 m Shuttle Radar Topography Mission (SRTM) Digital Elevation Model.
Annual Insolation	Relative potential incoming solar radiation (PISR). = PISR Winter solstice + PISR Summer solstice + PISR equinox (for methods see Boehner and Antoic 2009) Wetness Index a compound terrain attribute (sensu Bevan and Kirby 1979) implemented using the Shuttle Radar Topography Mission (SRTM) Digital Elevation Model and TOPOCROP Version 2.1 (Schmidt 2002)
Habitat Quality (Condition)	NVR2013_condV2

Mask Layers	Description
Native woody vegetation cover	Derived from NV2010_EXTENT_V2 from the Department of Environment and Primary Industries
Protected Areas	Derived from Public Land Management layer (PLM 25) updated 2013 from the Department of Environmental and Primary Industries. 1:25K polygons resampled to 75 m raster. Protected areas defined as any land-use type managed primarily for conservation.
National parks	Derived from 'Tourism Investment Opportunities in national parks Estate' from the Deparment of Environment and Primary Industries 2013
National Park Tourism Development Zones	Derived from 'Tourism Investment Opportunities in national parks Estate' from the Deparment of Environment and Primary Industries 2013

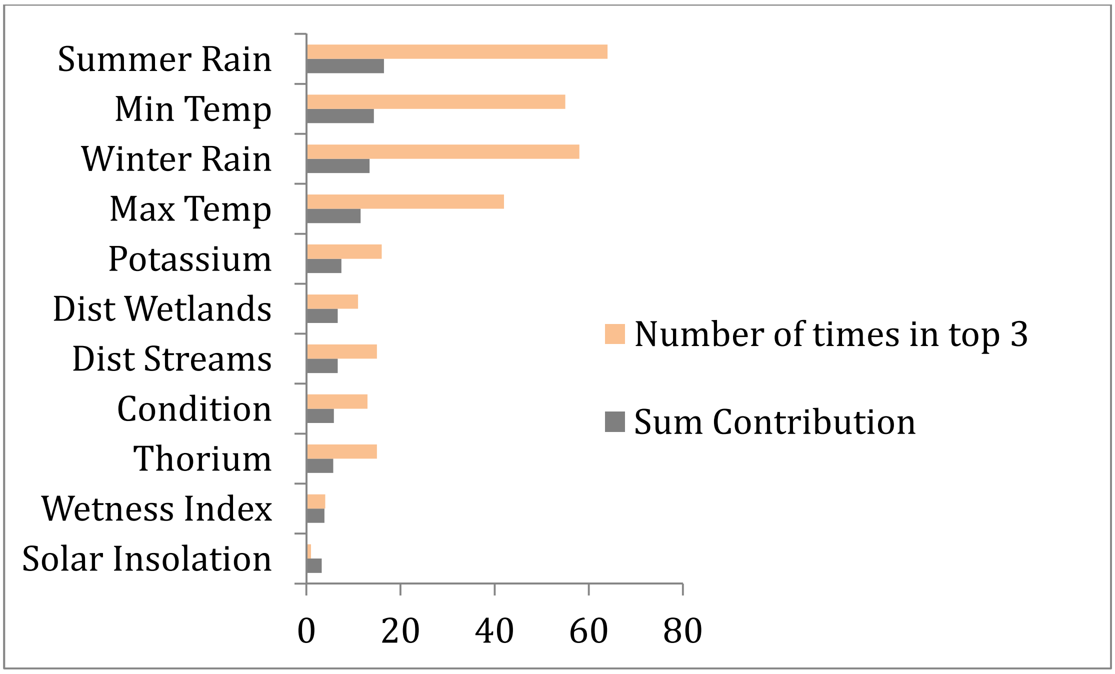

Figure S2 The overall contribution of each predictor variable represented by: (1) the number of times that particular predictor was one of the top three predictors for each individual model, and (2) the summed contribution of each variable calculated as the relative contribution of each variable to each model multiplied by the explanatory power of the model (AUC), then summed across all species models.

References

Beven, K.J., Kirkby, M.J. 1979. A physically-based variable contributing area model of basin hydrology' Hydrology Science Bulletin 24(1): 43-69.

Conrad, O. 2002. ChannelNetwork_Altitude.cpp, http://sourceforge.net/saga-gis: Accessed 24/05/2008.

Cook, S., Corner, R., Groves, P. & Grealish, G. 1996. Use of airborne gamma radiometric data for soil mapping. Australian Journal of Soil Research 34, 183-194.

Houlder, D., Hutchinson, M., Nix, H. & MacMahon, .J, 2001. ANUCLIM 5.1 Users Guide. The Australian National University, Center for Resource and Environmental Studies, Canberra.

Schmidt, F. and Persson, A. 2003. Comparison of DEM data capture and topographic wetness indices. Precision Agriculture 4:179-192.

Appendix S3: Eucalypt phylogeny with major taxonomic groups labeled: subgenera in black and sections within subg. Symphyomyrtus in grey. Posterior probabilities are labeled on nodes.

The *Corymbia* and *Angophora* clade is sister to genus *Eucalyptus.* Within the genus *Eucalyptus*, there are two subgenera in Victoria, subgenus *Eucalyptus* and subgenus *Symphyomyrtus*. Within subg. *Symphyomyrtus*, section Maidenaria and Exsertaria form a group, sections Adnataria, Dumaria, and part of Bisectae form a group, and the remainder of Bisectae forms a separate distinct group [1,2]. Subgenus *Eucalyptus* is less well resolved into section-level taxonomic categories [3]. Morphological data help differentiate some subg. *Eucalyptus* groups unresolved in ITS and ETS molecular phylogenies [4], but morphological data are not available for all species considered here.

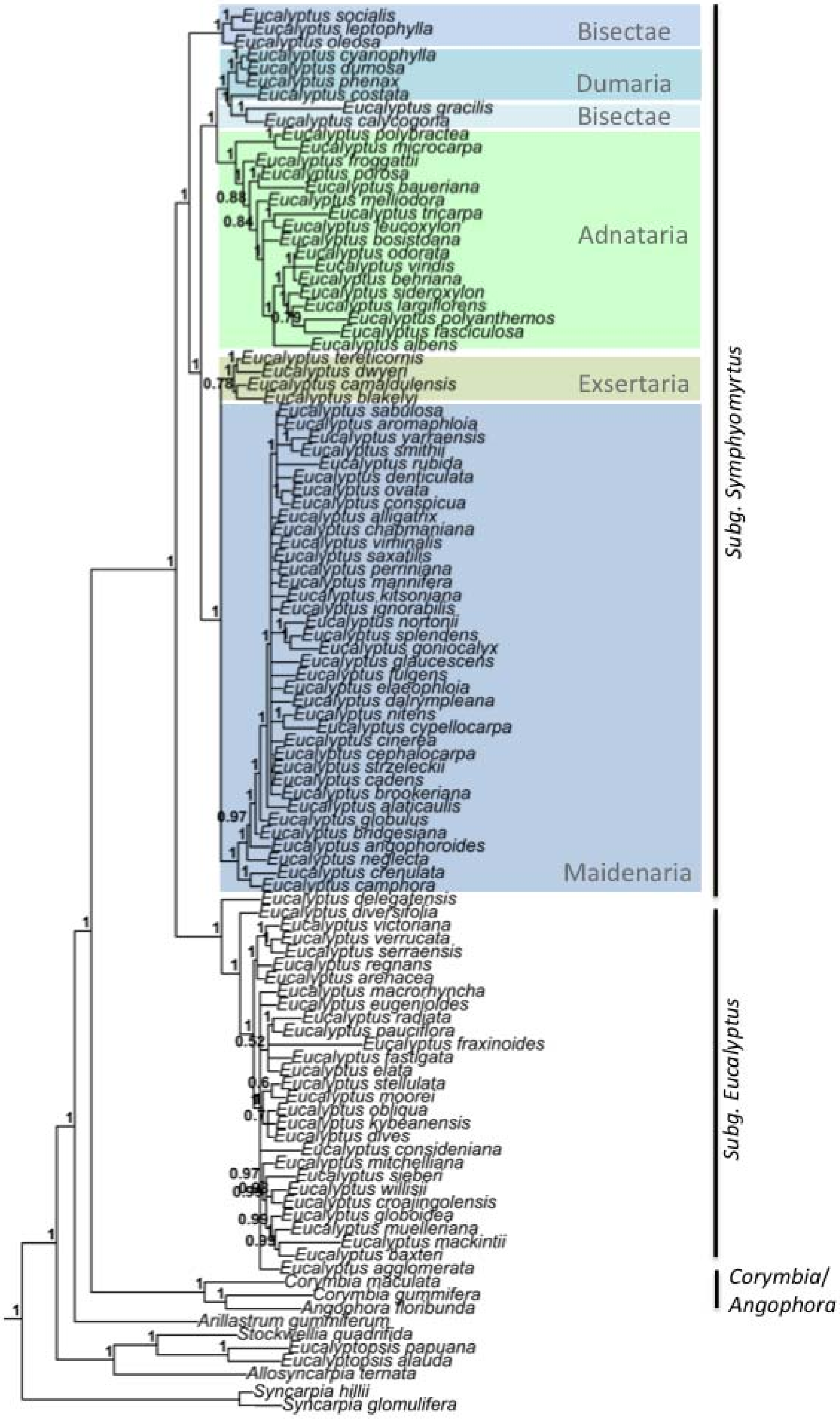
Eucalyptus socialis
Eucalyptus leptophylla
Eucalyptus oleosa
Bisectae
Eucalyptus cyanophylla
Eucalyptus dumosa
Eucalyptus phenax
Eucalyptus costata
Dumaria
Eucalyptus gracilis
Eucalyptus calycogona
Bisectae
Eucalyptus polybractea
Eucalyptus microcarpa
Eucalyptus froggattii
Eucalyptus porosa
Eucalyptus baueriana
Eucalyptus melliodora
Eucalyptus tricarpa
Eucalyptus leucoxylon
Eucalyptus bosistoana
Eucalyptus odorata
Eucalyptus viridis
Eucalyptus behriana
Eucalyptus sideroxylon
Eucalyptus largiflorens
Eucalyptus polyanthemos
Eucalyptus fasciculosa
Eucalyptus albens
Adnataria
Eucalyptus tereticornis
Eucalyptus dwyeri
Eucalyptus camaldulensis
Eucalyptus blakelyi
Exsertaria
Eucalyptus sabulosa
Eucalyptus aromaphloia
Eucalyptus yarraensis
Eucalyptus smithii
Eucalyptus rubida
Eucalyptus denticulata
Eucalyptus ovata
Eucalyptus conspicua
Eucalyptus alligatrix
Eucalyptus chapmaniana
Eucalyptus viminalis
Eucalyptus saxatilis
Eucalyptus perriniana
Eucalyptus mannifera
Eucalyptus kitsoniana
Eucalyptus ignorabilis
Eucalyptus nortonii
Eucalyptus splendens
Eucalyptus goniocalyx
Eucalyptus glaucescens
Eucalyptus fulgens
Eucalyptus elaeophloia
Eucalyptus dalrympleana
Eucalyptus nitens
Eucalyptus cypellocarpa
Eucalyptus cinerea
Eucalyptus cephalocarpa
Eucalyptus strzeleckii
Eucalyptus cadens
Eucalyptus brookeriana
Eucalyptus alaticaulis
Eucalyptus globulus
Eucalyptus bridgesiana
Eucalyptus angophoroides
Eucalyptus neglecta
Eucalyptus crenulata
Eucalyptus camphora
Maidenaria
Subg. Symphyomyrtus
Eucalyptus delegatensis
Eucalyptus diversifolia
Eucalyptus victoriana
Eucalyptus verrucata
Eucalyptus serraensis
Eucalyptus regnans
Eucalyptus arenacea
Eucalyptus macrorhyncha
Eucalyptus eugenioides
Eucalyptus radiata
Eucalyptus pauciflora
Eucalyptus fraxinoides
Eucalyptus fastigata
Eucalyptus elata
Eucalyptus stellulata
Eucalyptus moorei
Eucalyptus obliqua
Eucalyptus kybeanensis
Eucalyptus dives
Eucalyptus consideniana
Eucalyptus mitchelliana
Eucalyptus sieberi
Eucalyptus willisii
Eucalyptus croajingolensis
Eucalyptus globoidea
Eucalyptus muelleriana
Eucalyptus mackintii
Eucalyptus baxteri
Eucalyptus agglomerata
Subg. Eucalyptus
Corymbia maculata
Corymbia gummifera
Angophora floribunda
Corymbia/
Angophora
Arillastrum gummiferum
Stockwellia quadrifida
Eucalyptopsis papuana
Eucalyptopsis alauda
Allosyncarpia ternata
Syncarpia hillii
Syncarpia glomulifera

References

[1] McKinnon, G.E., Vaillancourt, R.E., Steane, D. & Potts, B.M. 2008 An AFLP marker approach to lower-level systematics in *Eucalyptus* (Myrtaceae). *American Journal of Botany* **95**, 368-380.

[2] Steane, D., Nicolle, D., McKinnon, G.E., Vaillancourt, R.E. & Potts, B.M. 2002 Higher-level relationships among the eucalypts are resolved by ITS-sequence data. *Aust. Syst. Bot.* **15**, 49-62.

[3] Brooker, M.I.H. 2000 A new classification of the genus *Eucalyptus* L'Hér. (Myrtaceae). *Aust. Syst. Bot.* **13**, 79-148.

[4] Ladiges, P.Y., Bayly, M.J. & Nelson, G.J. 2010 East-West Continental Vicariance in *Eucalyptus* Subgenus *Eucalyptus*. In *Beyond Cladistics: The Branching of a Paradigm* (eds. D.M. Williams & S. Knapp), pp. 267-302. Berkeley, University of California Press.

Appendix S4 Map of hypothetical expansion of the current protected area system by 5 percent (as in Fig. 3a) and 20 percent of their current area in blue. Expansion zones represent areas that best add to the PD that is already protected. A subset of this map is Figure 3b in the text.

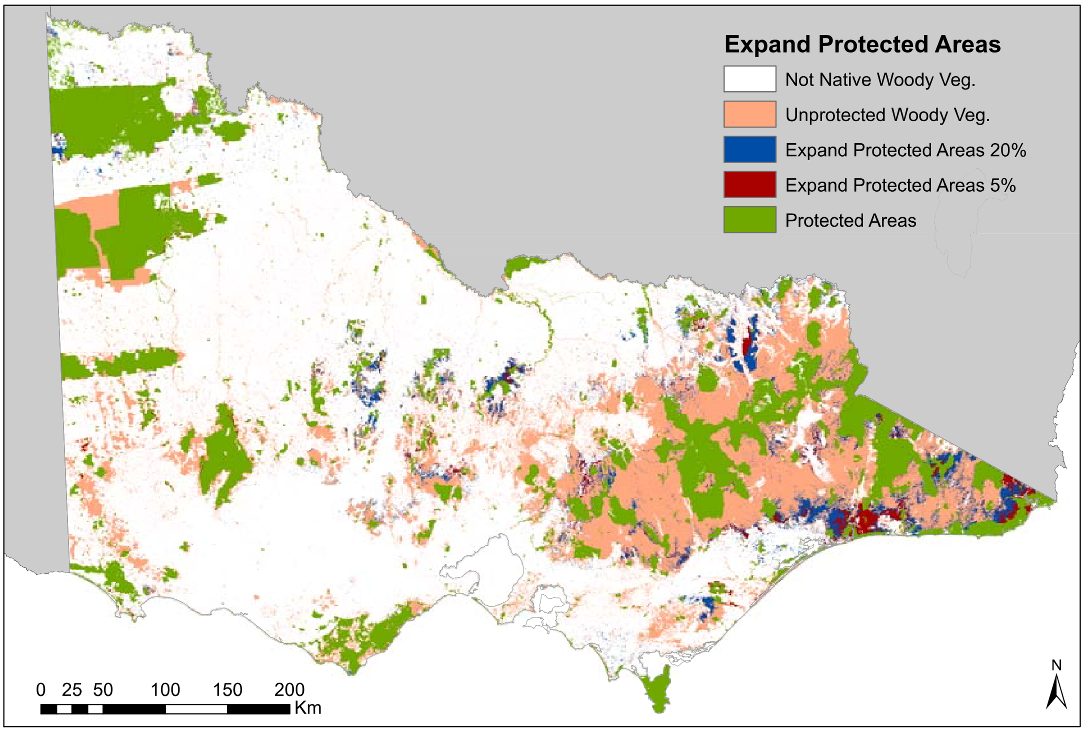

Appendix S5: Tourism Development in national parks Scenario for Victoria, Australia. The top 10 percent of tourism development zones ranked by PD contribution are shown as red. These development zones could be transferred to protected zones to capture 72% more PD than currently is found in protected zones.

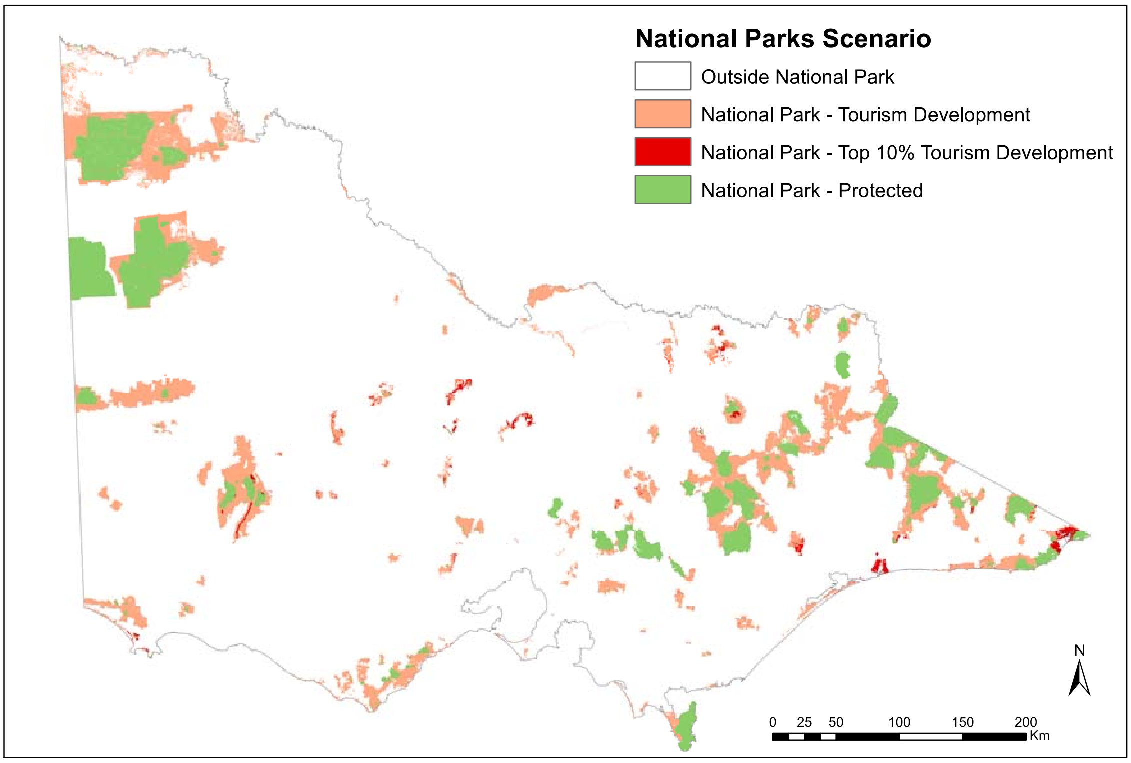

Supplement: Supplementary material [file rstb20140007supp.pdf]
